# Impact of lymph node retrieval on prognosis in elderly and non-elderly patients with T3-4/N+ rectal cancer following neoadjuvant therapy: a retrospective cohort study

**DOI:** 10.1007/s00384-024-04655-2

**Published:** 2024-06-06

**Authors:** Baofeng Liang, Sisi Xie, Nong Yu, Xueyi Xue, Hao Zeng, Zhipeng Que, Dongbo Xu, Xiaojie Wang, Shuangming Lin

**Affiliations:** 1 Department of Gastroenterology and Anorectal Surgery, Longyan First Affiliated Hospital of Fujian Medical University, No.105 Jiuyi North Road, Longyan, Fujian Province 364000 China; 2 Department of Colorectal Surgery, Union Hospital, Fujian Medical University, Fuzhou, People’s Republic of China; 3 Department of Surgery II, Shanghang Hospital, Longyan, China; 4 Department of Cardiology, Longyan First Affiliated Hospital of Fujian Medical University, Longyan, 364000 China

**Keywords:** Rectal cancer, Neoadjuvant therapy, Optimal lymph node interception values, The Surveillance, Epidemiology, and End Results (SEER), Survival prognosis

## Abstract

**Purpose:**

The optimal number of lymph nodes to be resected in patients with rectal cancer who undergo radical surgery after neoadjuvant therapy remains controversial. This study evaluated the prognostic variances between elderly and non-elderly patients and determined the ideal number of lymph nodes to be removed in these patients.

**Methods:**

The Surveillance, Epidemiology, and End Results (SEER) datasets were used to gather information on 7894 patients diagnosed with stage T3-4/N+ rectal cancer who underwent neoadjuvant therapy from 2010 to 2019. Of these patients, 2787 were elderly and 5107 were non-elderly. A total of 152 patients from the Longyan First Affiliated Hospital of Fujian Medical University were used for external validation. Overall survival (OS) and cancer-specific survival (CSS) were evaluated to determine the optimal quantity of lymph nodes for surgical resection.

**Results:**

The study found significant differences in OS and CSS between elderly and non-elderly patients, both before and after adjustment for confounders (*P* < 0.001). The removal of 14 lymph nodes may be considered a benchmark for patients with stage T3-4/N+ rectal cancer who undergo radical surgery following neoadjuvant therapy, as this number provides a more accurate foundation for the personalized treatment of rectal cancer. External data validated the differences in OS and CSS and supported the 14 lymph nodes as a new benchmark in these patients.

**Conclusion:**

For patients with T3-4/N+ stage rectal cancer who undergo radical surgery following neoadjuvant therapy, the removal of 14 lymph nodes serves as a cutoff point that distinctly separates patients with a favorable prognosis from those with an unfavorable one.

**Supplementary Information:**

The online version contains supplementary material available at 10.1007/s00384-024-04655-2.

## Introduction

Colorectal carcinoma (CRC) is one of the most common malignancies worldwide, characterized by high incidence and mortality. Up-to-date statistics from the USA show that, although the overall incidence of CRC is decreasing, an increasing number of cases are being diagnosed at an advanced stage. In addition, the incidence of CRC has increased in the younger population [1].

The management of advanced rectal cancer has changed dramatically over the past 40 years, thanks to new adjuvant and neoadjuvant therapies that complement surgical procedures. These therapies have transformed the approach to treatment, moving away from a traditional surgical approach to a more multifaceted and effective treatment method. Neoadjuvant therapy not only improves the success rate of radical surgery, but also effectively reduces local recurrence and increases the chance of sphincter preservation. Currently, the combination of neoadjuvant therapy and radical surgery is the standard treatment protocol for advanced rectal cancer [2].

In contrast to the declining incidence and mortality rates of CRC in patients over 50 years of age, an increase in incidence has been observed in the population under 50 years of age [3]. Five-year survival rates for CRC vary in patients of different ages [4], with patients over 60 years of age having lower contemporaneous survival rates than younger patients [5], highlighting the need for different management of CRC patients in different age groups.

Although the extent of local lymph node involvement is an important criterion in CRC staging and prognosis [6], there remains debate over the optimal number of lymph nodes to examine in patients with rectal cancer following neoadjuvant therapy. The American Joint Committee on Cancer (AJCC) [7] and the College of American Pathologists (CAP) recommend that at least 12 lymph nodes should be examined for precise staging of cancer [8]. For optimal lymph nodes examination, studies suggested that more examined lymph nodes are associated with better staging and survival [9]. Other research shows that retrieving fewer lymph nodes following preoperative chemoradiotherapy for rectal cancer does not necessarily mean that the surgery was incomplete [10]. It is clear that the accurate examination of lymph nodes plays a critical role in CRC staging. As a result, the determination of the ideal number of lymph nodes to be removed during radical surgery following neoadjuvant therapy is of significant clinical relevance.

This study was designed to investigate the optimal resection number of lymph nodes and to determine the risk factors affecting prognosis in patients with stage T3-4/N+ rectal cancer who received neoadjuvant therapy. We conducted extensive analyses of overall survival (OS) and cancer-specific survival (CSS) in elderly and non-elderly rectal cancer patients receiving neoadjuvant therapy. To verify the reliability of our findings, we validated these results with an external cohort. Our study utilized data from the Surveillance, Epidemiology and End Results (SEER) database.

## Materials and methods

### Included participants

This study used a retrospective cohort design with information on patients with single primary tumor T3-4/N+ rectal cancer selected from the SEER database of data from 2010 to 2019. Data were screened and extracted by using SEER*Stat software version 8.4.2. Inclusion criteria were as follows: (1) T3-4/N+ rectal cancer diagnosis and staging based on the TNM staging system, with tumor type and histological classification based on the International Classification of Diseases of Oncology, Third Edition (ICD-O-3); (2) diagnosis was made between 2010 and 2019; (3) there were valid follow-up data, and the cause of death of the deceased patients was confirmed; and (4) they received radical surgical treatment. Exclusion criteria: non-primary tumor patients, uncertain pathological diagnosis, follow-up data invalid, tumors in the appendix or an unknown location, and unclear pathological grading and tumor size. The number of lymph nodes is unclear, as is the grading of the tumor according to the AJCC (8th edition) was excluded from the study. For the purposes of this study, we collected information on each patient. This included diagnostic year, age of patients, sex, tumor stage and grade, size of the tumor, the total number of lymph nodes removed, extent of the regional lymph nodes, marriage status, carcinoembryonic antigen CEA pretreatment level, marital status, perineural infiltration (PNI), whether they had received postoperative chemotherapy or radiotherapy, tumor deposit, and the number of months they survived. Patients missing data on any variable will be excluded from the study. The reporting recommendations (STROBE) are as required by the Guidelines for Reporting.

### Data extraction

**Fig. 1:**
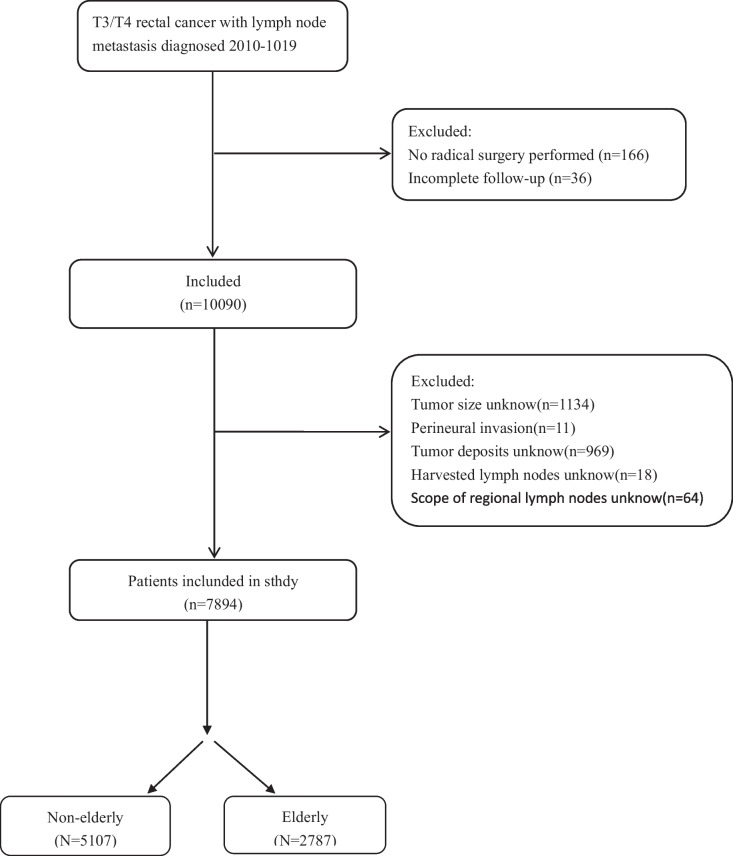
Patient cohort definition flowchart

In this study, we analyzed the data of 7894 patients. The marriage status was categorized as married and unmarried. Unmarried included those who were widowed, divorced, separated, or single. The results from the X-tile procedure were used to categorize the number of lymph nodes removed (nLN) into two groups: ≥ 12 and < 12. Additionally, tumor size was classified as ≥ 5 cm and < 5 cm (Fig. 1). To validate the study model, data were collected from a group of patients with T3-4/N+ rectal cancer from the Longyan First Affiliated Hospital of Fujian Medical University. The study was conducted in accordance with the principles outlined in the Declaration of Helsinki and received ethical approval from the Ethics Committee of the Longyan First Affiliated Hospital of Fujian Medical University under approval number LYREC2024-k028-01.

To mitigate potential sources of bias in this paper, we conducted rigorous data screening in the SEER database during data collection to ensure that the data was diverse, comprehensive, and balanced. We also made efforts to avoid biases in data collection. During data preprocessing, we cleaned and screened the database to remove outliers and duplicate data, among other operations, to avoid interference in the analysis results. Finally, we validated the model through external verification to confirm its feasibility.

### Statistical analysis

This study aimed to evaluate two primary survival endpoints: cancer-specific survival (CSS) and overall survival (OS). CSS was defined as the duration from the date of diagnosis to death caused by metastasis, recurrence, or related factors, or until the conclusion of the follow-up period. OS was defined as the duration from the patient’s date of diagnosis to death from any cause. The study focused on CSS, while OS served as a secondary outcome. The authors compared categorical variables using Fisher’s exact test and continuous variables using *t*-tests. Survival curves for OS and CSS were estimated using the Kaplan-Meier method, with between-group differences assessed using log-rank tests. Univariate and multivariate Cox proportional risk models were used to explore prognostic factors associated with OS and CSS. To address the issue of unequal baseline data, the authors employed propensity score matching (PSM), which allowed them to balance various important covariates, including age, race, tumor grade, tumor location, number of harvested lymph nodes, extent of regional lymph nodes examined, marital status, preoperative CEA level, tumor size, and marital status. All statistical analyses were conducted using R software (version 4.3.1).

## Results

### Patient characteristics

A total of 7894 patients diagnosed with T3-4/N+ rectal cancer who were receiving neoadjuvant therapy were divided into two groups: the elderly patient group (*n* = 2787) and the non-elderly patient group (*n* = 5107). The baseline characteristics of the two groups of patients are presented in Table 1. Compared to the non-elderly group, the elderly group had significantly higher (*P* < 0.05) tumor stage, pN, proportion receiving chemotherapy, pre-treatment CEA level, number of lymph nodes resected, proportions of tumor size < 5 cm, and peripheral nerve invasion (PNI). To adjust for potential confounders between patient groups, we applied a propensity score matching (PSM) method; Table 2 shows the baseline characteristics after matching. After matching, the two groups showed no significant differences (*P* > 0.05) in all the factors studied, suggesting that propensity score matching effectively balanced the baseline characteristics between the two groups.

**Table 1:** Baseline characteristics

	**Non-elderly (*N* = 5107)**	**Elderly (*N* = 2787)**	***P*-value**
**Sex**			
Female	1948 (38.1%)	1075 (38.6%)	0.726
Male	3159 (61.9%)	1712 (61.4%)	
**Scope of regional lymph nodes**			
1 to 3 regional lymph nodes	151 (3.0%)	107 (3.8%)	0.0901
4 or more regional lymph nodes	4813 (94.2%)	2596 (93.1%)	
No/unknown	143 (2.8%)	84 (3.0%)	
**Histopathology**			
Adenocarcinoma	4843 (94.8%)	2612 (93.7%)	0.079
Mucinous adenocarcinoma	231 (4.5%)	158 (5.7%)	
Signet ring cell carcinoma	33 (0.6%)	17 (0.6%)	
**Stage**			
IIA	1943 (38.0%)	1301 (46.7%)	< 0.001
IIB	52 (1.0%)	35 (1.3%)	
IIC	153 (3.0%)	89 (3.2%)	
IIIB	2415 (47.3%)	1159 (41.6%)	
IIIC	544 (10.7%)	203 (7.3%)	
**pT**			
T3	4485 (87.8%)	2493 (89.5%)	0.0729
T4a	173 (3.4%)	89 (3.2%)	
T4b	449 (8.8%)	205 (7.4%)	
**pN**			
N0	2148 (42.1%)	1425 (51.1%)	< 0.001
N1	673 (13.2%)	301 (10.8%)	
N1a	769 (15.1%)	406 (14.6%)	
N1b	709 (13.9%)	326 (11.7%)	
N1c	135 (2.6%)	83 (3.0%)	
N2a	416 (8.1%)	155 (5.6%)	
N2b	257 (5.0%)	91 (3.3%)	
**Chemotherapy**			
No/unknown	61 (1.2%)	90 (3.2%)	< 0.001
Yes	5046 (98.8%)	2697 (96.8%)	
**CEA pretreatment**			
CEA negative/normal	2131 (41.7%)	1098 (39.4%)	0.00187
CEA positive/elevated	1732 (33.9%)	909 (32.6%)	
Unknow	1244 (24.4%)	780 (28.0%)	
**Harvested lymph nodes**			
< 14LNs	2080 (40.7%)	1336 (47.9%)	< 0.001
≥ 14LNs	3027 (59.3%)	1451 (52.1%)	
**Harvested lymph nodes**			
< 12LNs	1234 (24.2%)	877 (31.5%)	< 0.001
≥ 12LNs	3873 (75.8%)	1910 (68.5%)	
**Tumor size**			
< 5 cm	3996 (78.2%)	2256 (80.9%)	0.00515
≥ 5 cm	1111 (21.8%)	531 (19.1%)	
**Tumor deposits**			
No/unknown	4631 (90.7%)	2539 (91.1%)	0.562
Yes	476 (9.3%)	248 (8.9%)	
**Perineural invasion**			
No/unknown	4416 (86.5%)	2493 (89.5%)	< 0.001
Yes	691 (13.5%)	294 (10.5%)	
**Marital status**			
Married	2983 (58.4%)	1684 (60.4%)	0.0846
Unknown	175 (3.4%)	107 (3.8%)	
Unmarried	1949 (38.2%)	996 (35.7%)

**Table 2:** Baseline characteristics after propensity score matching

	**Non-elderly (*N* = 2732)**	**Elderly (*N* = 2732)**	***P*-value**
**Sex**			
Female	1050 (38.6%)	1048 (38.5%)	0.978
Male	1669 (61.4%)	1671 (61.5%)	
**Scope of regional lymph nodes**			
1 to 3 regional lymph nodes	102 (3.8%)	99 (3.6%)	0.977
4 or more regional lymph nodes	2535 (93.2%)	2538 (93.3%)	
No/unknown	82 (3.0%)	82 (3.0%)	
**Histopathology**			
Adenocarcinoma	2554 (93.9%)	2557 (94.0%)	0.984
Mucinous adenocarcinoma	150 (5.5%)	147 (5.4%)	
Signet ring cell carcinoma	15 (0.6%)	15 (0.6%)	
**Stage**			
IIA	1248 (45.9%)	1257 (46.2%)	0.938
IIB	32 (1.2%)	33 (1.2%)	
IIC	79 (2.9%)	88 (3.2%)	
IIIB	1164 (42.8%)	1141 (42.0%)	
IIIC	196 (7.2%)	200 (7.4%)	
**pT**			
T3	2446 (90.0%)	2431 (89.4%)	0.658
T4a	75 (2.8%)	86 (3.2%)	
T4b	198 (7.3%)	202 (7.4%)	
**pN**			
N0	1359 (50.0%)	1378 (50.7%)	0.965
N1	304 (11.2%)	297 (10.9%)	
N1a	412 (15.2%)	399 (14.7%)	
N1b	323 (11.9%)	323 (11.9%)	
N1c	83 (3.1%)	82 (3.0%)	
N2a	159 (5.8%)	150 (5.5%)	
N2b	79 (2.9%)	90 (3.3%)	
**Chemotherapy**			
No/unknown	47 (1.7%)	41 (1.5%)	0.591
Yes	2686 (98.3%)	2685 (98.3%)	
**CEA pretreatment**			
CEA negative/normal	1110 (40.8%)	1079 (39.7%)	0.603
CEA positive/elevated	888 (32.7%)	890 (32.7%)	
Unknown	721 (26.5%)	750 (27.6%)	
**Harvested lymph nodes**			
< 14LNs	1270 (46.7%)	1295 (47.6%)	0.514
≥ 14LNs	1449 (53.3%)	1424 (52.4%)	
**Harvested lymph nodes**			
< 12LNs	788 (29.0%)	841 (30.9%)	0.124
≥ 12LNs	1931 (71.0%)	1878 (69.1%)	
**Tumor size**			
< 5 cm	2206 (81.1%)	2298 (80.8%)	0.809
≥ 5 cm	513 (18.9%)	521 (19.2%)	
**Tumor deposits**			
No/unknown	2484 (91.4%)	2477 (91.1%)	0.774
Yes	235 (8.6%)	242 (8.9%)	
**Perineural invasion**			
No/unknown	2425 (89.2%)	2430 (89.4%)	0.861
Yes	294 (10.8%)	289 (10.6%)	
**Marital status**			
Married	1596 (58.7%)	1648 (60.6%)	0.18
Unknown	94 (3.5%)	105 (3.9%)	
Unmarried	1029 (37.8%)	966 (35.5%)	

### Survival analysis in non-elderly and elderly T3-4/N+ rectal cancer patients

**Fig. 2:**
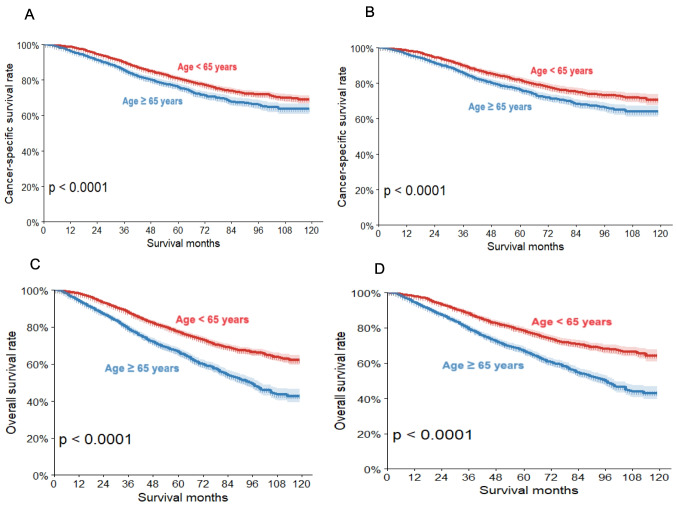
Comparison of survival between the non-elderly group and the elderly group. **A** CSS of non-matched patients; **B** OS of non-matched patients; **C** CSS of propensity score matching patients; **D** OS of propensity score matching patients. OS, overall survival; CSS, cancer-specific survival

Before PSM, The non-elderly group had higher cancer-specific survival (CSS) and overall survival (OS) rates than the elderly group, (CSS 83.8% vs. 80.0%, respectively, *P* < 0.001; OS 80.2% vs. 68.1%, *P* < 0.001) (Fig. 2A, C). After PSM, CSS and OS rates remained; the elderly group had a lower percentage than the non-elderly group (CSS 80.3% vs. 83.8%, *P* < 0.001; OS 68.6% vs. 80.1%, *P* < 0.001) (Fig. 2B, D).

### Analysis of optimal number of lymph nodes removed and survival rates

**Fig. 3:**
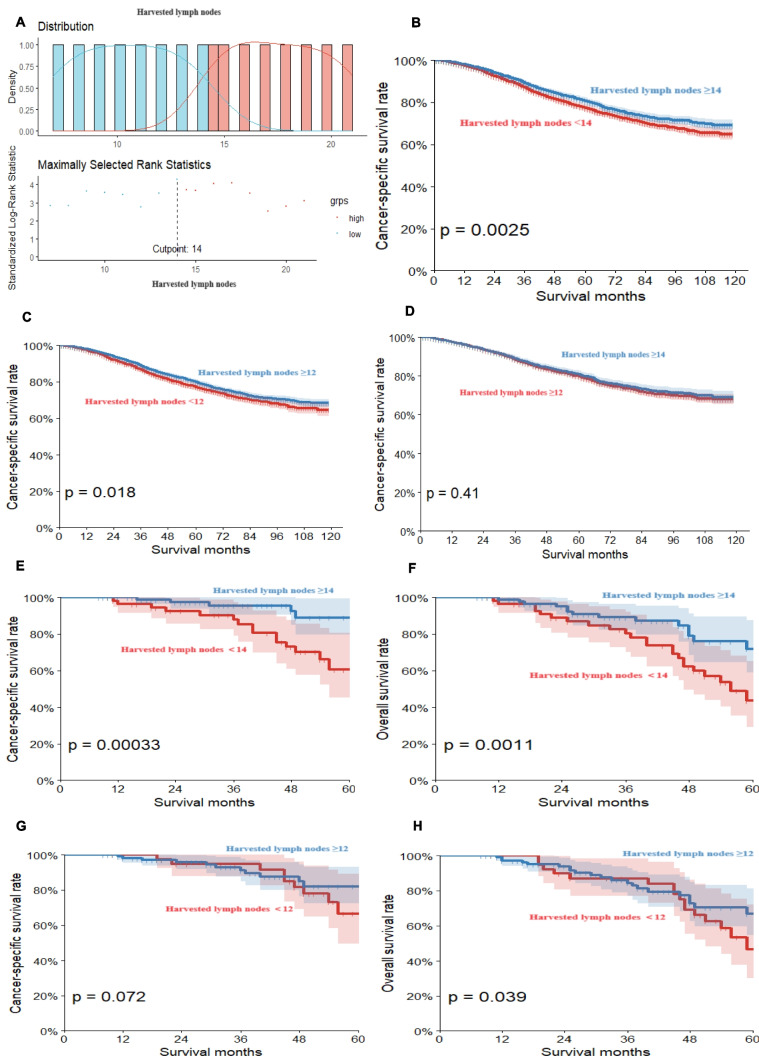
Optimal number of lymph nodes resected and associated cancer-specific and overall survival in T3-T4/N+ rectal cancer after neoadjuvant therapy. The optimal threshold for lymph node resection was as follows: The number of lymph nodes resected was 14. **B**, **C** Comparison of CSS: lymph node ≥ 14 vs. < 14 lymph nodes group and lymph node ≥ 12 vs. < 12 lymph nodes group, respectively. **D** Comparison of CSS for lymph node ≥ 12 and ≥ 14 groups. **E**, **F** Validation cohorts: comparison of CSS and OS in ≥ 14 lymph nodes vs. < 14 lymph nodes. **G**, **H** Validation cohort: CSS and OS comparison of ≥ 12 lymph nodes versus < 12 lymph nodes

This study, which analyzed patients with T3-4/N+ rectal cancer after neoadjuvant therapy using the survminer R package, identified 14 lymph nodes as the optimal threshold for resection (Fig. 3A). The survival analysis clearly demonstrated that patients with 14 or more resected lymph nodes had a significantly higher CSS than those with fewer than 14 lymph nodes resected (86.0% vs. 80.7%, *P* < 0.005, Fig. 3B). The conventional threshold of 12 lymph nodes is clear: patients with 12 or more had a higher chance of survival than those with less than 12 (85.5% vs. 78.8%, *P* < 0.05, Fig. 3C). There was no significant difference observed in cancer-specific survival (CSS) between the group with 14 or more lymph nodes removed and the group with 12 or more lymph nodes removed (*P* = 0.41, Fig. 3D). To validate these findings, an additional 152 patients from the Longyan First Affiliated Hospital of Fujian Medical University were utilized for external validation. The validation analysis confirmed that ≥ 14 lymph nodes significantly improved CSS and OS compared with < 14 lymph nodes. In contrast, no significant difference was found in OS or CSS when comparing patients with ≥ 12 lymph nodes to those with < 12 lymph nodes (Fig. 3E–H). Therefore, we are certain that the 14 lymph node threshold is a better way of distinguishing patients with better prognoses from those with poor prognoses.

### Analyzed using univariate and multivariate Cox regression models

We conducted univariate and multivariate analyses to identify significant prognostic factors for cancer-specific survival (CSS) in rectal cancer patients. Our findings suggest that age of patients, sex, histopathology, pT stage, pN stage, extent of regional lymph nodes examined, presence of tumor deposits, pretreatment CEA level, total number of lymph nodes removed, receipt of chemotherapy, perineural invasion (PNI), tumor size ≥ 5 cm, and marriage status were all significantly correlated with worse CSS (each *P* < 0.05). These factors may serve as useful predictors for the prognosis of rectal cancer patients (Table 3). Furthermore, age of patients, sex, pN, chemotherapy, pretreatment CEA level, the total number of lymph nodes removed ≥ 14, PNI, tumor deposits, and marriage status were all found to be independent prognostic factors for CSS.

**Table 3:** Univariate and multivariable analysis of factors associated with cancer-specific survival

**Variable**	**Univariate analysis**				**Multivariate analysis**			
	HR	95%CI		P-value	HR	95%CI		P-value
**Age**								
< 65 years	Reference				Reference			
≥ 65 years	1.33	1.19	1.48	< 0.001	1.47	1.31	1.64	< 0.001
**Sex**								
Female	Reference				Reference			
Male	1.074	1.002	1.150	0.044	1.26	1.12	1.40	< 0.001
**Histopathology**								
Adenocarcinoma	Reference				Reference			
Mucinous adenocarcinoma	1.60	1.32	1.93	< 0.001	1.28	1.06	1.56	0.012
Signet ring cell carcinoma	3.95	2.66	5.87	< 0.001	3.08	2.07	4.60	< 0.001
**pT**								
T3	Reference				Reference			
T4a	1.11	0.79	1.56	0.553	0.99	0.65	1.50	0.956
T4b	2.10	1.79	2.47	< 0.001	1.52	0.93	2.49	0.097
**pN**								
N0	Reference				Reference			
N1	0.88	0.50	1.54	0.658	1.04	0.48	2.21	0.929
N1a	1.52	1.30	1.77	< 0.001	1.54	0.90	2.64	0.113
N1b	1.85	1.59	2.15	< 0.001	1.85	1.08	3.17	0.026
N1c	2.83	2.19	3.66	< 0.001	1.97	1.10	3.53	0.022
N2a	2.28	1.91	2.72	< 0.001	2.37	2.39	4.04	0.002
N2b	3.86	3.21	4.65	< 0.001	3.62	1.94	4.46	< 0.001
**Scope of regional lymph nodes**								
1 to 3 regional LNs	Reference				Reference			
4 or more regional LNs	0.75	0.59	0.96	0.024	0.89	0.68	1.16	0.392
None	0.59	0.40	0.89	0.012	0.68	0.45	1.03	0.072
**Chemotherapy**								
No/Unknown	Reference				Reference			
Yes	0.54	0.40	0.74	< 0.001	0.56	0.41	0.76	< 0.001
**CEA Pretreatment**								
CEA negative/normal	Reference				Reference			
CEA positive/elevated	1.61	1.42	1.83	< 0.001	1.38	1.21	1.57	< 0.001
Unknow	1.29	1.13	1.48	< 0.001	1.18	1.03	1.35	0.02
**Harvested lymph nodes**								
< 12LNs	Reference				Reference			
≥ 12LNs	0.87	0.78	0.98	0.018	0.97	0.83	1.14	0.709
**Harvested lymph nodes**								
< 14LNs	Reference				Reference			
≥ 14LNs	0.85	0.77	0.94	0.003	0.79	0.68	0.91	0.002
**Tumor deposits**								
No	Reference				Reference			
Yes	2.21	1.91	2.56	< 0.001	1.36	1.15	1.60	< 0.001
**Tumor size**								
< 5 cm	Reference				Reference			
≥ 5 cm	1.00	1.00	1.01	< 0.001	1.08	0.97	1.2	0.164
**Perineural invasion**								
No/Unknown	Reference				Reference			
Yes	2.57	2.26	2.91	< 0.001	2.06	1.81	2.36	< 0.001
**Marital status**								
Married	Reference				Reference			
Unknow	1.13	0.85	1.51	0.402	1.15	0.86	1.54	0.338
Unmarried	1.29	1.16	1.44	< 0.001	1.29	1.16	1.44	< 0.001

### Subgroup analyses for resected lymph nodes and CSS in elderly and non-elderly groups

**Fig. 4:**
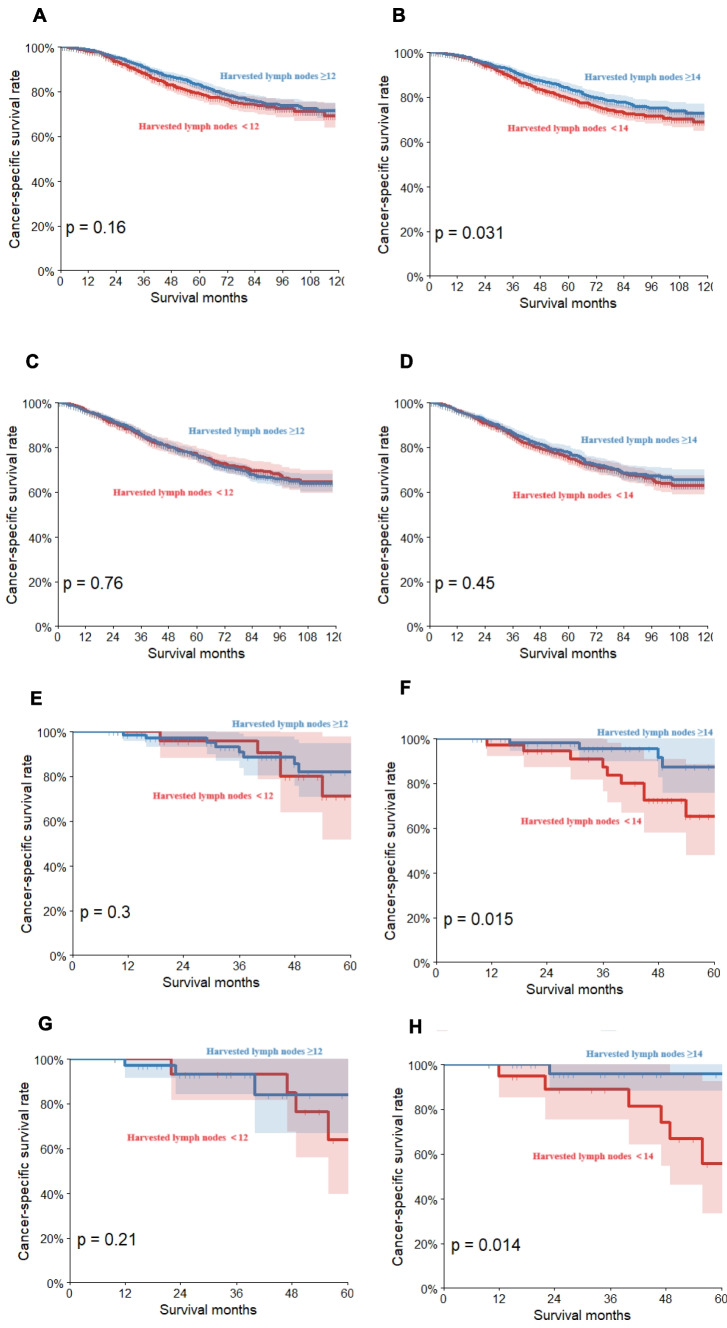
Subgroup analysis of harvested lymph nodes and cancer-specific survival (CSS) in elderly and non-elderly groups: **A**, **B** non-elderly: CSS for ≥ 12 vs. < 12 lymph nodes and ≥ 14 vs. < 14 lymph nodes. **C**, **D** Elderly: CSS for ≥ 12 vs. < 12 lymph nodes and ≥ 14 vs. < 14 lymph nodes. **E**, **F** Validation non-elderly: CSS for ≥ 12 vs. < 12 lymph nodes and ≥ 14 vs. < 14 lymph nodes. **G**, **H** Validation elderly: CSS for ≥ 12 vs. < 12 lymph nodes and ≥ 14 vs. < 14 lymph nodes

We further explored the influence of the resected number of lymph nodes on cancer-specific survival (CSS) in both the elderly and non-elderly groups. Our analyses revealed a significant association between the number of resected lymph nodes and CSS in both groups (Fig. 4A–H). In our external validation cohort, we explored the potential impact of the specific location of lymph nodes, chemotherapy regimen, radiotherapy course, and microsatellite status on reaching the optimal threshold of 14 resected lymph nodes by subgroup analysis. The results of the analyses showed a significant difference between resection of at least 14 lymph nodes and survival in the group of patients with negative N0.253 lymph nodes *(P* < 0.05). However, in other subgroups, no significant association was observed between reaching the threshold of 14 lymph nodes and survival, regardless of the specific location of the lymph nodes, chemotherapy regimen, course of radiotherapy, or microsatellite status. Thus, the removal of more lymph nodes appears to be associated with better survival. Our findings suggest that other factors, such as treatment regimen or pathological features, have insignificant impact on the resected lymph nodes reaching this threshold (Supplementary file 1A-N).

## Discussion

Colorectal cancer (CRC) is a common gastrointestinal malignancy that represents a major health threat, given its high morbidity and mortality rates. It is increasingly impacting younger populations and often presents at advanced stages [1]. Surgery-based combination therapy including neoadjuvant radiotherapy is the standard treatment option for colorectal cancer, especially for locally progressive low and intermediate rectal cancers, which is of great significance for anus-preservation, R0 resection, and survival of locally progressive rectal cancer patients [11]. The number of examined lymph nodes is crucial for pathological staging and has been strongly associated with survival outcomes. In rectal cancer, the adequate removal of lymph nodes is an essential indicator of the quality of radical rectal cancer surgery [12]. While both the National Comprehensive Cancer Network (NCCN) and the American Joint Committee on Cancer (AJCC) recommend examining at least 12 lymph nodes after surgery for CRC [13], this recommendation is primarily based on patients who did not receive neoadjuvant therapy and underwent radical surgical resection as their initial treatment. However, neoadjuvant therapy can induce a regressive response in lymph nodes similar to that of the primary tumor, making postoperative lymph node detection more challenging [14]. Moreover, a lower number of identified lymph nodes after neoadjuvant therapy does not imply genuine reduced lymph nodes. Guillaume Chotard et al. found that preoperative neoadjuvant therapy for locally advanced rectal cancer did not change the number of lymph nodes or the number of positive lymph nodes [15]. The optimal number of resected lymph nodes for patients receiving neoadjuvant therapy must be determined. There is considerable variation in the number of examined lymph nodes among rectal cancer patients who receive neoadjuvant therapy, with values ranging from 4 to 14 per sample, according to previous studies [14, 16, 17]. In the current study, the optimal number of resected lymph nodes is 14. This may be closely related to the standardized treatment and routine performance of high lymph node ligation at the root of the inferior mesenteric artery at our center.

In our study, patients with resected lymph node counts greater than 14 had better OS and CSS than those with less than 14. The number of examined lymph nodes is an indicator of the quality of surgery and risk stratification and subsequently guides adjuvant therapy for patients diagnosed with stage II or III CRC. Accurate lymph node assessment is crucial for pathological staging, subsequent treatment following total mesorectal excision (TME), and prognosis [18]. Previous studies have investigated the number of lymph nodes in patients undergoing radical surgery for rectal cancer. A higher number of detected lymph nodes may affect staging, quality of surgery, and quality of pathological specimens, thus affecting survival in these patients [19]. In our study, the number of detected lymph nodes emerged as an independent prognostic factor. More extensive lymph node retrieval may lead to a more favorable prognosis. However, Hong Yang et al. [20] definitively demonstrated that a lymph node count of fewer than 12 is not a predictor of poor prognosis. This study combined data from the SEER database and data from our center and concluded that a lymph node count of 14 was associated with improved OS and CSS. These observations are supported by this conclusion.

In this study, “elderly” refers to individuals aged 65 years and above. It is important to note that the characteristics of malignancies, including CRC, in elderly individuals differ from those observed in younger populations [5, 21–23]. There is an absence of studies that examine the impact of lymph node count following neoadjuvant therapy on the prognosis of elderly versus non-elderly patients with CRC. In the current study, among patients with more than 14 resected lymph nodes who underwent radical surgery for rectal cancer after neoadjuvant therapy, those younger than 65 years had better OS and CSS outcomes than older patients. This may be related to decreased physical strength and weakness in older patients after surgery.

It is widely acknowledged that age negatively affects lymph node sampling [24–26]. The number of samples decreases by 9% for every 10 years of age [26]. Giovanni Li Destri hypothesized that in older patients, the number of samples would be affected due to physiological deterioration of the lymph nodes, a weakened immune response, and the presence of comorbidities [27]. This is consistent with Restivo A’s findings, which demonstrated that age is a risk factor for early distant recurrence in rectal cancer patients who received preoperative radiotherapy [28]. The results of our study indicated that age was an independent prognostic factor, as demonstrated by univariate and multivariate analyses.

The number of lymph nodes detected is indicative of the quality of surgical radical treatment in the case of rectal cancer and is also an essential factor for the assessment of risk stratification and guidance of subsequent adjuvant therapy for patients with stage II and stage III rectal cancer [29]. For patients undergoing neoadjuvant radiotherapy, the number of lymph nodes detected can provide a more intuitive reflection of the efficacy of the neoadjuvant therapy. In general, a reduction in tumor size was negatively correlated with the number of lymph nodes acquired. Overall, the number of lymph nodes obtained is typically less than 12, which has been the subject of debate regarding the rate of local recurrence and distant metastasis [8, 30]. Chase J Wehrle et al. have concluded that the criteria for detecting 12 lymph nodes after neoadjuvant chemoradiation therapy (nCRT) and neoadjuvant therapy are not consistent in resectable stage III rectal cancer. The requirement for lymph node harvest (LNH) for pathological lymph node staging may vary depending on the tumor’s response to neoadjuvant treatment [31]. Matthew D. Hall et al. suggested that at least eight lymph nodes should be examined in patients with rectal cancer undergoing nCRT, noting that eight lymph nodes represent the threshold for adequate lymph node dissection after nCRT [32]. Xu Guan et al. [33] demonstrated that an increased number of LNH was associated with more accurate lymph node staging and a higher survival rate. Another study concluded that 15 LNH represented the optimal threshold for the assessment of the quality of lymph node examination and prognostic stratification [10]. Our study indicated that individuals with greater than 14 lymph nodes excised demonstrated superior OS and CSS outcomes. These findings are warranted to be further validated.

Several limitations of this study should be noted: Firstly, the lack of comprehensive data on the specific location and pathological examination methods in the SEER database precluded their inclusion in our multifactorial analysis. Secondly, the lack of information on adjuvant therapy indications, treatment regimens, and chemotherapy cycles precludes any assessment of the relationship between lymph node count and adjuvant therapy. Thirdly, the external validation sample employed in this study was derived from a Chinese center, which represents a limited sample size. Finally, the study center and the SEER database did not provide specific data regarding the waiting period after radiotherapy. It is necessary to include this variable in future studies to more accurately evaluate its impact on treatment outcomes.

## Conclusion

This study unequivocally demonstrates the importance of retrieving lymph nodes during radical surgery for rectal cancer following neoadjuvant therapy. For patients diagnosed with stage T3-4/N+ rectal cancer undergoing radical surgery after neoadjuvant therapy, 14 lymph nodes represent a new threshold for prognostic differentiation. Although the difference in lymph node retrieval between counts of 12 and 14 was not statistically significant, using a benchmark of 14 lymph nodes more accurately distinguished patients with favorable prognoses from those with less favorable outcomes compared to a benchmark of 12 lymph nodes.

## Supplementary Information

Below is the link to the electronic supplementary material.

Supplementary file1: A hierarchical analysis was performed on T3-4/N+ rectal cancers based on the optimal number of 14 lymph nodes removed. The analysis was stratified by the location of the lymph nodes (A-H), the chemotherapy regimen (I, J), the course of radiotherapy (K, L), and the microsatellite status (M, N). (PDF 248 KB)

## Data Availability

No datasets were generated or analyzed during the current study.
