# Supplementary material for: Impact of lymph node retrieval on prognosis in elderly and non-elderly patients with T3-4/N+ rectal cancer following neoadjuvant therapy: a retrospective cohort study
Source: Int J Colorectal Dis. 2024 Jun 6;39(1):86. doi: 10.1007/s00384-024-04655-2 (PMC11156732; doi:10.1007/s00384-024-04655-2)
Supplement: Supplementary file 1 — Supplementary file1: A hierarchical analysis was performed on T3-4/N+ rectal cancers based on the optimal number of 14 lymph nodes removed. The analysis was stratified by the location of the lymph nodes (A-H), the chemotherapy regimen (I, J), the course of radiotherapy (K, L), and the microsatellite status (M, N). (PDF 248 KB) [file 384_2024_4655_MOESM1_ESM.pdf]

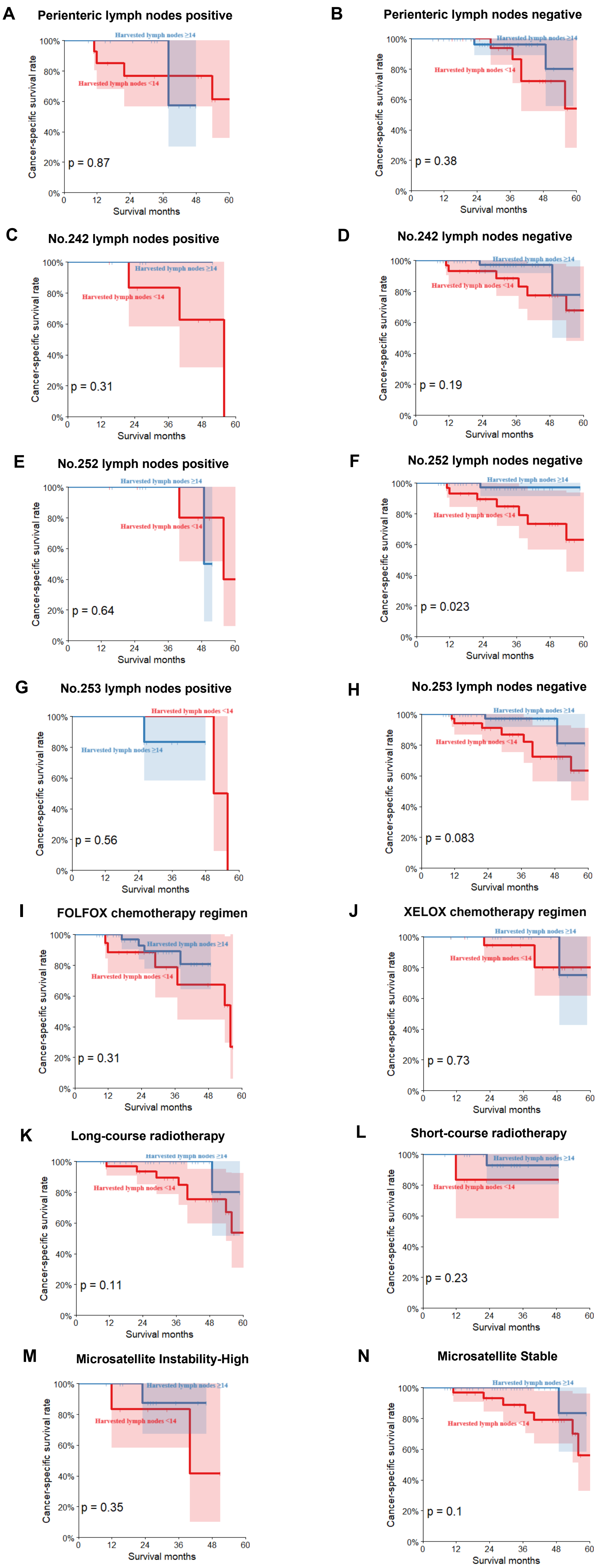

A
Perienteric lymph nodes positive
Harvested lymph nodes ≥14
Harvested lymph nodes <14
p = 0.87
B
Perienteric lymph nodes negative
Harvested lymph nodes ≥14
Harvested lymph nodes <14
p = 0.38
C
No.242 lymph nodes positive
Harvested lymph nodes ≥14
Harvested lymph nodes <14
p = 0.31
D
No.242 lymph nodes negative
Harvested lymph nodes ≥14
Harvested lymph nodes <14
p = 0.19
E
No.252 lymph nodes positive
Harvested lymph nodes ≥14
Harvested lymph nodes <14
p = 0.64
F
No.252 lymph nodes negative
Harvested lymph nodes ≥14
Harvested lymph nodes <14
p = 0.023
G
No.253 lymph nodes positive
Harvested lymph nodes <14
Harvested lymph nodes ≥14
p = 0.56
H
No.253 lymph nodes negative
Harvested lymph nodes ≥14
Harvested lymph nodes <14
p = 0.083
I
FOLFOX chemotherapy regimen
Harvested lymph nodes ≥14
Harvested lymph nodes <14
p = 0.31
J
XELOX chemotherapy regimen
Harvested lymph nodes ≥14
Harvested lymph nodes <14
p = 0.73
K
Long-course radiotherapy
Harvested lymph nodes ≥14
Harvested lymph nodes <14
p = 0.11
L
Short-course radiotherapy
Harvested lymph nodes ≥14
Harvested lymph nodes <14
p = 0.23
M
Microsatellite Instability-High
Harvested lymph nodes ≥14
Harvested lymph nodes <14
p = 0.35
N
Microsatellite Stable
Harvested lymph nodes ≥14
Harvested lymph nodes <14
p = 0.1
Cancer-specific survival rate
0% 20% 40% 60% 80% 100%
Survival months
0 12 24 36 48 60
